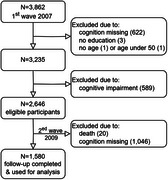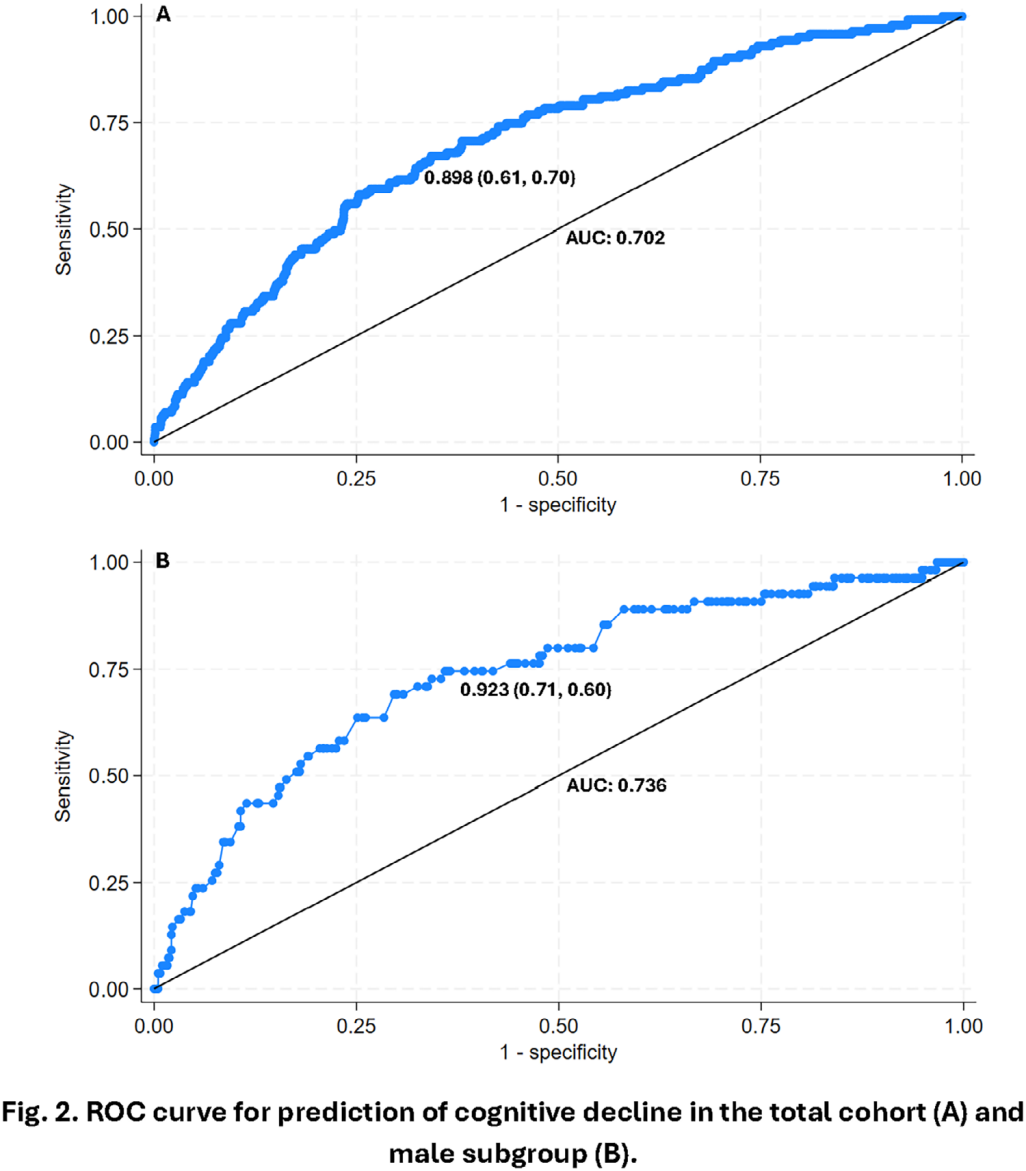# Memory function combined with grip strength predicts cognitive decline among older Japanese men: A prospective study

**DOI:** 10.1002/alz70861_108396

**Published:** 2025-12-23

**Authors:** Lihui Tu, Yasuo Kuniyoshi

**Affiliations:** ^1^ RIKEN, Wakoshi, Saitama Japan

## Abstract

**Background:**

Memory function impairment is often an early manifestation of cognitive aging and dementia, especially in rapidly aging populations such as Japan. Identifying risk factors and predictors for cognitive decline is essential for early intervention. We aimed to investigate the predictive factors of cognitive decline among community‐dwelling older Japanese adults.

**Method:**

This study included 1,580 cognitively healthy individuals aged 50–75 years at baseline, drawn from the Japanese Study of Aging and Retirement between 2007 and 2009. Memory function was assessed using immediate and delayed word recall tasks, with scores ranging from 0 to 20. Cognitive decline was defined as a 2009 memory score lower than the baseline mean minus 1 SD, adjusted for education level. Variables related to demographics, health, lifestyle, cognition, and emotion were included. Multivariable logistic regression analyses were conducted to identify predictors of cognitive decline, and receiver operating characteristic (ROC) analysis was performed to assess model performance.

**Result:**

Over the 2‐year follow‐up, 146 participants (9.2%) developed memory cognitive decline. Older age, male gender, former smoking, frequent alcohol consumption, a lower baseline memory score, and weaker grip strength were associated with an increased risk of cognitive decline. In the multivariable‐adjusted model, female gender (OR = 0.34, 95% CI: 0.20–0.58), higher baseline memory score (OR = 0.77, 95% CI: 0.71–0.84), and stronger grip strength (OR = 0.97, 95% CI: 0.94–1.00) were associated with a lower risk of cognitive decline, with an area under the ROC curve (AUC) of 0.702. In the subgroup analysis of males (n = 847), a model including baseline memory (OR = 0.72, 95% CI: 0.62–0.85) and grip strength (OR = 0.92, 95% CI: 0.87–0.97) achieved an AUC of 0.736 for predicting cognitive decline. No significant predictive performance was observed among females (AUC < 0.70).

**Conclusion:**

Combining memory function and grip strength offers a promising approach for predicting cognitive decline among community‐dwelling older Japanese men. These simple and non‐invasive measures may help identify individuals at higher risk, supporting the development of targeted prevention strategies.